# Case of Rupture of the Uterus in the Third Month of Pregnancy

**Published:** 1826-07

**Authors:** 


RUPTURE OF THE UTERUS IN EARLY PREGNANCY.

Messrs. Moulin and Guibert presented to the Academy of Medicine, in the late autumn, a piece of morbid anatomy, of which we shall here give the history, somewhat abbreviated.

Mad. Cayer, 26 years of age, had laid in of her first child, at full term, in the year 1821. This event was succeeded by two miscarriages, one at four, and the other at 5 months, in the years 1823 and 1825. Her health had not suffered from these accidents, and she again became pregnant in May, 1825. Nothing particular occurred until the 7th of August, when, having taken a very hot bath and dined lightly, she repaired to an assembly, where she amused herself with dancing, of which she was passionately fond. In the midst of a waltz, she felt something crack in the lower part of the abdomen, and instantly fell down in a state of syncope. She was conveyed to bed, and the most alarming symptoms rapidly supervened. The abdomen swelled and became painful—the pulse faultered—the countenance sank—the body was covered with cold sweats—inexpressible anxiety and restlessness came on, with vomiting, &c. Messrs. Moulin and Guibert were quickly on the spot; and, on examination, no discharge from the vagina was discovered, nor any thing unusual about the uterus. One of the medical attendants came to the conclusion, that a rupture of the *liver* had taken place. Twenty leeches were applied to the right hypochondrium, followed by fomentations, acidulated diluents, &c. At four o'clock in the morning, the medical gentlemen were again summoned, as the patient was said to be dying. More physicians were called in, and though all agreed that the poor lady was at the point of death, there was great discordance, as usual, in respect to the cause of the fatal catastrophe. The scalpel cleared up the mystery. There was nothing wrong in the head or chest. On laying open the abdomen, a quantity of pure and fluid blood flowed out. The liver and other abdominal organs were all sound. Attached to the uterus was seen a round mass of coagulated blood, in the centre of which was found a fœtus of about ten weeks old, encircled with its membranes. There was a rent in the fundus of the uterus, through which the fœtus had escaped into the abdominal cavity.

Around this rupture the substance of the womb appeared rather softened, but not thinner than in any other part. There was no other morbid phenomenon worthy of record.

This is certainly a most rare, if not a unique case of rupture of the uterus:—For we do not recollect any instance of the kind on record, at so early a period of pregnancy. But a suggestion was started by one of the gentlemen at the academy when the parts were examined there, that the case was probably one of those which Carus calls *demi-extra-uterine* pregnancies—that is, where the fœtus is developed in the parietes, instead of the cavity of the uterus, in the vicinity of the opening of a fallopian tube—a species of extra-uterine impregnation so accurately described by M. Breschet. Would the Cæsarian section, at an early period after the rupture, have offered any chance of success, with or without transfusion of blood?—*Archives*, 1825.

